# Pharmacokinetic Pharmacodynamic Modelling Contributions to Improve Paediatric Anaesthesia Practice

**DOI:** 10.3390/jcm11113009

**Published:** 2022-05-26

**Authors:** James D. Morse, Luis Ignacio Cortinez, Brian J. Anderson

**Affiliations:** 1 Department of Anaesthesiology, University of Auckland, Park Road, Auckland 1023, New Zealand; j.morse@auckland.ac.nz; 2 División Anestesiología, Escuela de Medicina, Pontificia Universidad Católica de Chile, San Diego de Chile 8331150, Chile; icortinez@gmail.com

**Keywords:** anaesthetics, intravenous, paediatrics, anaesthetic techniques, TIVA, target-controlled infusion, pharmacokinetics, pharmacodynamics, nonlinear mixed-effects models

## Abstract

The use of pharmacokinetic-pharmacodynamic models has improved anaesthesia practice in children through a better understanding of dose-concentration-response relationships, developmental pharmacokinetic changes, quantification of drug interactions and insights into how covariates (e.g., age, size, organ dysfunction, pharmacogenomics) impact drug prescription. Simulation using information from these models has enabled the prediction and learning of beneficial and adverse effects and decision-making around clinical scenarios. Covariate information, including the use of allometric size scaling, age and consideration of fat mass, has reduced population parameter variability. The target concentration approach has rationalised dose calculation. Paediatric pharmacokinetic-pharmacodynamic insights have led to better drug delivery systems for total intravenous anaesthesia and an expectation about drug offset when delivery is stopped. Understanding concentration-dependent adverse effects have tempered dose regimens. Quantification of drug interactions has improved the understanding of the effects of drug combinations. Repurposed drugs (e.g., antiviral drugs used for COVID-19) within the community can have important effects on drugs used in paediatric anaesthesia, and the use of simulation educates about these drug vagaries.

## 1. Introduction

Pharmacokinetic-pharmacodynamic (PKPD) modelling has contributed to our understanding of drugs used in children for both anaesthesia and analgesia [1,2]. While data used to model can be difficult to collect in children, population modelling allows those data to be pooled from multiple separate studies, sampling time windows rather than strict times can be employed, and children with missing data points do not have to be excluded from the analysis.

Simulation using PKPD models can facilitate the understanding and prediction of exposure-response relationships in children, demonstrate effects of drug interactions, estimate dose, balance beneficial effects over adverse effects, test hypotheses, ascertain biological plausibility and quantify variability.

This review describes aspects of PKPD modelling used in children and demonstrates the application of recent paediatric modelling to clinical practice.

## 2. Models

Population PKPD modelling is a statistical approach in which mathematical equations are used to describe typical time-concentration and concentration-response relationships observed after drug administration. These equations define a model that is used to describe the relationship observed. Compartment models commonly describe anaesthesia drug disposition within the body. Time–concentration relationships for a one-compartment pharmacokinetic model, for example, are expressed in terms of the parameters clearance (CL) and volume of distribution (V):

(1) Concentration =doseV × e−time × CLV

Drugs used in anaesthesia are not usually confined to one compartment. Two- and three-compartment models are more common. These are still parameterised in terms of clearance and volume, but a three-compartment model may have a central volume (V1) and two peripheral volumes (V2, V3), while clearance (CL) also comprises inter-compartment clearances (Q2, Q3). Computers have made the estimation of these parameters easier. Nonlinear regression methods directly estimate parameters through iterative techniques using least-squares curve fitting. Models with more than a single compartment are now invariably solved using differential equations rather than graphical techniques.

A description of the drug effect may require an additional compartment (the effect compartment) to account for time delays between plasma concentration and observed response. The versatile Hill equation [3] is used in many areas of physiology (e.g., oxygen dissociation) and pharmacology (e.g., protein binding, Michaelis-Menten kinetics). It is commonly used to relate drug concentration (C) to response (or effect):

(2) $$\mathrm{Response}=E_{\mathrm{MAX}}\;\frac{C^{\mathrm{HILL}}}{C_{50}^{\;\mathrm{HILL}}+{\;C}^{\mathrm{HILL}}}$$

The pharmacodynamic parameter, E_MAX_, is the maximum drug effect, C_50_ is the concentration eliciting half of E_MAX_, and the Hill exponent describes the steepness of the concentration-response curve. The concentration used to describe the observed response can be in the effect compartment (Ce) rather than the plasma (Cp).

## 3. Key Elements of Paediatric PKPD Models

Children differ from adults, and the two main areas of diversity are growth and development and size; these are important sources of variability. The primary aim of clinical pharmacology is the prediction of drugs PK and PD in the individual patient, and this is achieved by acquiring knowledge of the covariates that contribute to variability [4].

Body composition changes with age. Size is commonly standardised using weight, while age is used as a surrogate for growth and development. Pharmacokinetic parameter estimates and the use of a target concentration allow the dose to be determined by accounting for changes that occur with covariates such as size and age, drug interactions, pharmacogenomics, and organ dysfunction—concerns about adverse effects temper dose.

### 3.1. Size

When clearance is expressed as per kilogram per unit of time, it is greater at 1–2 years of age and declines in older children and adolescents. This high clearance after maturation can be attributed to the nonlinear relationship between size and function and is explained using the allometric theory [5]. The use of allometric size scaling rather than per-kilogram size scaling results in a clearance in children over 2 years old that is the same as in adults [6]. Maturation changes are generally completed within the first 2 years of postnatal life; consequently, babies may be considered immature children, whereas children are just small adults [7]. Pharmacokinetic parameters (e.g., clearance, CL; volume, V; half-time, T_1/2_) can be standardised (F_SIZE_) to a typical adult with total body weight (TBW) of 70 kg using allometric scaling [8,9].

(3) FSIZE=TBWCHILD70EXP

The allometric exponent (EXP) is ¾ for clearance, 1 for volume and ¼ for half-time. The allometric exponent of ¾ for clearance has been widely used for intravenous drugs in paediatric anaesthesia, e.g., propofol [10,11], morphine [12], methadone [13], remifentanil [14,15], oxycodone [16], dexmedetomidine [17], rocuronium [18], caffeine [19], and acetaminophen [20]. The allometric theory has also been used to determine sevoflurane compartment pharmacokinetics in children, allowing the estimation of gas uptake and elimination and an effect-site equilibration half-time (T_1/2_keo) [21]. When scaled using the allometric theory, this half-time is smaller in children than in adults, contributing to the observed more rapid onset of effect.

### 3.2. Age

The clearance of most drugs increases over the first years of life [22]. This increase is usually described using postmenstrual age (PMA) to account for clearance maturation that begins in utero. Clearance maturation is commonly described using a maturation function (MF), another variant of the Hill equation [3]:

(4) $$\mathrm{MF}=\frac{{\mathrm{PMA}}^{\mathrm{HILL}}}{{\mathrm{TM}}_{50}^{\;\mathrm{HILL}}+{\mathrm{PMA}}^{\mathrm{HILL}}}$$

The TM_50_ describes the maturation half-time, while the Hill exponent relates to the slope of this maturation profile. The parameter (P, e.g., clearance or volume) can be described:

(5) $$P_{\mathrm{CHILD}}={\;P}_{\mathrm{ADULT}\;}\times F_{\mathrm{SIZE}}\times \mathrm{MF}$$

This standardisation of size and maturation has proven popular [6] and is useful for learning about biology, comparing different studies, detecting errors and rationalizing dose prediction. The use of this standard approach to size and maturation supports a consistent method of dosing in patients of all ages [1,2,23,24]. This approach forms the basis for the universal or general-purpose models [10,14] that are used in target-controlled infusion pumps. Such models accommodate individuals that range from neonates to the elderly across a wide range of weights undergoing total intravenous anesthesia [25].

### 3.3. Body Composition

Preterm and term neonates have a smaller proportion of body weight in the form of fat and muscle mass compared with children and adolescents. With growth, the proportion of body weight composed of these tissues increases and total body water decreases [26]. A well-reported consequence of this is that neuromuscular blocking drugs, which are distributed to extracellular fluid, have a greater volume of distribution in infants. A bigger loading dose (expressed as per kilogram) is required to achieve the desired target concentration and clinical response [27].

### 3.4. Obesity

Obesity among children and adolescents in the United States has increased threefold in the past 30 years, with approximately one in seven children and adolescents estimated to be obese [28]. There are few published guidelines for anaesthesia drug dosing in obese children [29,30], creating a quandary for clinicians [31]. 

Volume (determining loading dose) and clearance (determining maintenance dose) of some drugs are known to be changed in obesity [32]. The effect of fat mass on clearance is probably minimal; it has minimal metabolic activity. It does, however, contribute to body size, and body size is used as a scaler for clearance. The volume of distribution of a drug depends partially on its physicochemical properties [33]. Unfortunately, these properties may be poor markers for volume scaling. Some drugs have an apparent distribution volume independent of fat mass (e.g., digoxin); others are extensively determined by fat mass (e.g., diazepam). A number of size descriptors have been put forward for use in obese patients. Total body weight (TBW) remains the commonest, but others have been proposed that might better account for fat mass influence, e.g., fat-free mass (FFM), lean body weight (LBW), body mass index (BMI), ideal body weight (IBW), and normal fat mass (NFM) [34]. The issue is further confused because the size metric used for a loading dose (determined by volume) may differ from that used for a maintenance dose or infusion (determined by clearance) [34].

Ideal body weight has been proposed as the preferred metric for maintenance dosing of benzodiazepines (diazepam [35], midazolam [36]), morphine [37] and neuromuscular blocking drugs such as vecuronium [38], rocuronium [39,40], and cisatracurium [41]. Lean body weight (often used interchangeably with fat-free mass) is believed to be the optimal size scaler for other drugs, including some opioids and anaesthetic induction agents [30,42,43,44]. The use of LBW appears to be a suitable size scaler for remifentanil clearance [45].

### 3.5. Normal Fat Mass

Normal fat mass is a size descriptor that uses fat-free mass plus a ‘bit more’. The ‘bit more’ will differ for each drug, and the maximum ‘bit more’ added to the fat-free mass would equal TBW [46]. The use of normal fat mass (NFM) [47] with allometric scaling as a size descriptor gives versatility to this descriptor [48,49].

(6) $$\mathrm{NFM}=\mathrm{FFM}+\mathrm{Ffat}\;\times \left(\mathrm{TBW}-\mathrm{FFM}\right)$$

The ‘bit more’ is determined by the parameter Ffat. When estimated, it can be used to account for different contributions of fat mass to clearance or volume. If Ffat is estimated to be zero, then FFM alone predicts size. When Ffat is predicted to be one, then size is predicted by TBW. This parameter will differ for each drug. A negative value for Ffat might suggest organ dysfunction, not an uncommon scenario in the morbidly obese. A negative value has been reported for dexmedetomidine in that cohort [50].

Fat mass does have a contribution to dexmedetomidine volume (Ffat = 0.293), but has little contribution to clearance (Ffat = 0) [17]. Total body weight (Ffat = 1) can be used for propofol [51], fentanyl [52] and oxycodone [16]. Normal fat mass was the best covariate to describe acetaminophen clearance, with a factor for fat contribution (Ffat) of 0.816 [53]. The concept has also been applied to physiological functions. It has a value of 0.211 for the glomerular filtration rate, which implies that 21% of fat mass is a size driver for kidney function in addition to FFM [48].

## 4. Target Concentration Concepts

The goal of treatment is the target effect. A pharmacodynamic (PD) model (e.g., the Hill equation) is used to predict the target concentration known to be associated with a target effect.

### 4.1. Achieving the Target Effect

Population pharmacokinetic-pharmacodynamic parameter estimates and covariate information are used to predict time-concentration and concentration-effect values in a specific patient. For example, a dexmedetomidine steady-state target concentration of 0.6 µg/L may be the target concentration in children managed in an intensive care unit after cardiac surgery. The dose will increase from an infusion of 0.33 µg/kg/h in a neonate to 0.51 µg/kg/h in a 1-year-old child because the clearance matures over the first year of life. A lower dose of 0.47 µg/kg/h in an 8-year-old is used because of size factors [54]. An infusion duration of 4 half-lives is required to reach 93.74% of the steady-state concentration. Consequently, a loading dose is used to achieve the target concentration rapidly. Rapid infusion, however, can be associated with adverse cardiovascular effects.

The target concentration strategy is extremely useful for determining the clinical dose [55]. This approach is used almost instinctively by paediatric anaesthetists using target-controlled infusion systems. These devices target a specific plasma or effect-site concentration in a typical individual, and this concentration is assumed to have a typical target effect. Covariates such as the patient’s age or size are manually entered into the TCI pump program. Adverse effects are monitored (e.g., hypotension). The target concentration is one that achieves a target therapeutic effect (e.g., anesthesia) without excessive adverse effects. Subsequent monitoring of drug concentrations in serum and Bayesian forecasting may be used to improve the dose in individual patients. This facility is not yet available for use with TCI pumps, but the ability to determine propofol concentration from expired breath holds promise [56].

### 4.2. Defining Target Concentration

An effect-site target concentration has been estimated for many drugs used in anesthesia, analgesia, and sedation. The relationship between propofol concentration and effect (bispectral index, BIS) using the Hill equation [57] has been used to identify a target concentration of 3 mg/L (3 µg/mL) that achieves a BIS of 40–60. A BIS monitor provides feedback to guide the propofol infusion rate to achieve the desired target effect rather than the target concentration in children and teenagers. Neuromuscular monitoring provides a similar feedback system for neuromuscular blocking drugs. Unfortunately, feedback systems are not available for most drugs. The depth of anaesthesia monitors is of little value in neonates and infants where neither the target concentration is known nor the electroencephalogram monitoring adequate [11].

Concentration-response relationships for clonidine [58], sevoflurane [59] and dexmedetomidine [60,61] have recently been established, enabling target concentration estimation. New drugs should have a concentration-response relationship described early in the drug development process [62,63]. This relationship has been described for alfaxalone, a new intravenous induction agent [64], facilitating further focussed studies as part of drug development. Unfortunately, concentration-effect information is missing for many older drugs.

## 5. Practical Applications

Pharmacokinetics and pharmacodynamics are the sciences that supply information for dose determination. The parameters used in models (PK: CL, V; PD: E_MAX_, C_50_) to describe PKPD relationships are associated with variability. Adverse effects related to concentration may temper the target concentration. This section will describe examples of different clinical scenarios where PKPD modelling can be used to improve dosing regimens and understand the reasons for those regimens.

### 5.1. Using Variability for Dose Estimation

An understanding of the magnitude of variability is important for dose determination and toxicity concerns. Neuromuscular blocking drugs, for example, must be given at a dose that is effective for every patient. The dose may be as much as twice or three times as effective in 95% (ED_95_) of children [18]. Concentrations after levobupivacaine caudal injection should be lower than those associated with toxicity, but that concentration should be lower in all children. Simulation has been used to investigate safe doses using parameter estimates and their variability [65]. Total levobupivacaine plasma concentrations increase during a 48 h epidural infusion, attributable to increases in the reactive alpha-1 acid glycoprotein after surgical insult. Protein binding models have been used to demonstrate that unbound levobupivacaine concentrations remain at steady-state and do not contribute to increased toxicity [66].

Oral morphine administered regularly after tonsillectomy is associated with a range of measured concentrations. The quantification of that range allows the estimation of a dose that is known to contribute to concentrations lower than those causing respiratory depression [67,68].

### 5.2. Improving Inhalational Agent Use

The pharmacokinetics of inhaled anaesthetics is mostly based on physiology [69]. Pharmacodynamics are usually described using minimal alveolar concentration (MAC).

#### 5.2.1. Integrated PKPD for Inhaled Drugs

The relationship between sevoflurane expired concentration and bispectral index (BIS, a measure of drug effect) has been described using compartment models and a sigmoid E_MAX_ response in children [21] and adults [70]. Size standardisation using allometry adequately explained clearance and volume changes with age. The effect-site concentration eliciting half the maximum response (Ce_50_) at the age of 40 years was 1.3 vol%, and that decreased with age from 1.6 vol% at 3 years to 1.1 vol% at 70 years, consistent with reports of similar changes with age when investigated using minimal alveolar concentration (MAC). The equilibration half-time (T_1/2_keo 1.48 min) could be predicted using allometry in those younger than 40 years [21,71].

The use of compartment models, similar to those employed for intravenous anaesthetic drugs, has the potential to improve understanding of interactions between intravenous and inhalational agents, particularly when using hybrid techniques with both inhalational and total intravenous anaesthesia (TIVA). This modelling technique will also allow closer simultaneous examination of physiological covariate effects (e.g., cardiac output, respiratory function) on the dynamics of inhalational agents.

#### 5.2.2. Sevoflurane Induction, Propofol Maintenance

Propofol TIVA in children can start after gaseous induction with sevoflurane once intravenous access is obtained. Using a fixed infusion rate of propofol after a gaseous induction may be inadequate because steady-state concentrations will not be established before 3–4 elimination half-lives. Reports of awareness have occurred after the switch from gaseous inhalation anaesthesia to TIVA, particularly when a loading dose was not given or when that loading dose was delayed [72]. The small loading dose of propofol required remains uncertain, but 1 mg/kg is believed satisfactory [73].

**Figure 1:**
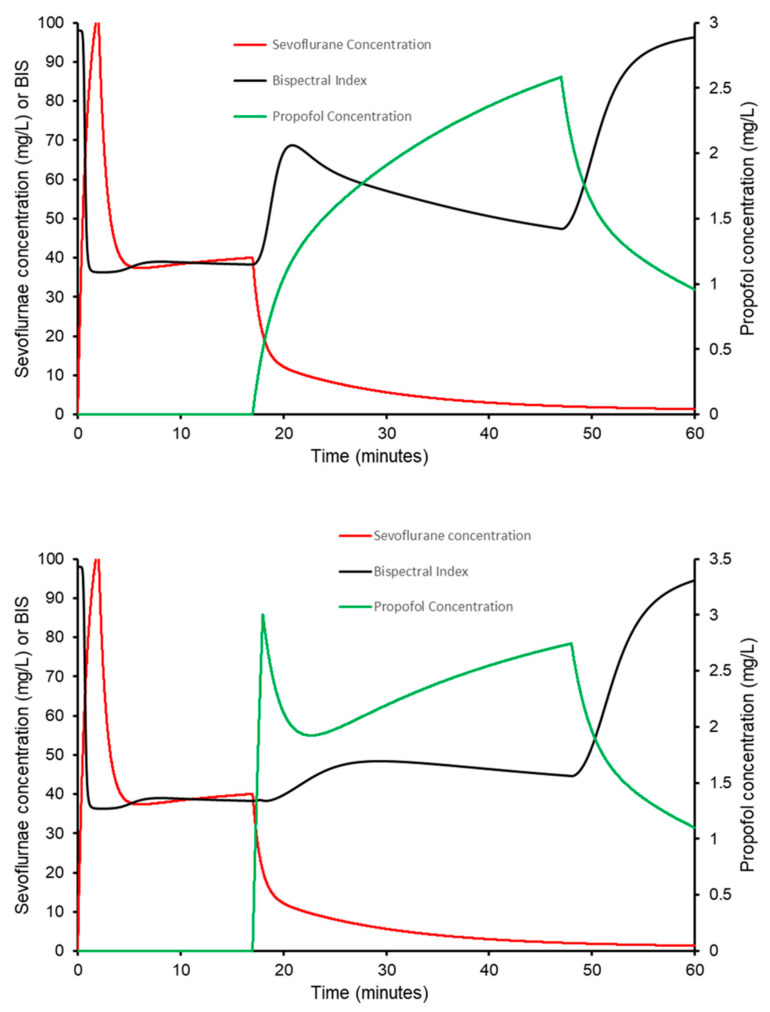
Inhalational induction with 8% sevoflurane was followed after 2 min with 2% inhaled sevoflurane to maintain a bispectral index of 40. Cessation of sevoflurane caused a rapid increase in the bispectral index if only a propofol infusion of 10 mg/h/kg was administered (upper panel). That BIS was maintained at an acceptable score (40–60) if a modest dose of propofol 1 mg/kg was given before propofol infusion.

Simulation using a sevoflurane compartment model with propofol-sevoflurane interaction [74] has been used to portray these concepts (Figure 1). Inhalational induction with 8% sevoflurane was followed with 2% inhaled sevoflurane to maintain a bispectral index of 40. Cessation of sevoflurane caused a rapid increase in the bispectral index if only a propofol infusion of 10 mg/h/kg was started. That BIS was maintained at an acceptable score (40–60) if a modest dose of propofol 1 mg/kg was given before propofol infusion.

### 5.3. Improving Propofol Intravenous Anaesthesia

There are few practical recommendations for propofol infusion in neonates and infants. This is attributable to a scarcity of pharmacokinetic parameter estimates in this cohort, a cohort undergoing physiological maturation. The lower age bracket recommended in common target-controlled infusion (TCI) devices is 1–3 years [75,76,77] because pharmacokinetics are poorly described in children.

#### 5.3.1. Propofol Target-Controlled Infusion

A “universal” pharmacokinetic-pharmacodynamic (PKPD) parameter set that is applicable to neonates, infants and children is reported [78]. Propofol time-concentration and bispectral index (BIS) data were analysed from 1033 individuals of a wide age range (27 weeks postmenstrual age to 88 years). Growth and maturation aspects of propofol disposition were incorporated into these estimated parameters. However, the assessment of covariates such as fat mass and the use of BIS as a surrogate electroencephalographic (EEG) measure of the depth of anaesthesia remains uncertain in neonates and infants [34,46]. The uncertainty of processed EEG devices in neonates and infants makes identification of a target concentration difficult.

#### 5.3.2. Manual Infusion

Manual infusion regimens for propofol have been suggested based on rich pooled data in neonates, infants and children [11]. A dosing regimen predicted to achieve a steady-state concentration of 3 mg/L required a loading dose of 2 mg/kg followed by an infusion rate of 9 mg/kg/h for the first 15 min, 7 mg/kg/h from 15 to 30 min, 6 mg/kg/h from 30 to 60 min, and 5 mg/kg/h from 1 to 2 h in neonates (38–44 weeks postmenstrual age). The dose was bigger in those aged 1–2 years, consistent with per-kilogram clearance maturation. Propofol clearance increased throughout infancy to reach 92% of that reported in adults (1.93 L/min/70 kg) by 6 months postnatal age. The authors identified a target propofol concentration of 3 mg/L, but the target concentration is unknown in neonates and may be less than 3 mg/L. Consequently, dosing regimens for a lower target of 2 mg/L were presented [11].

#### 5.3.3. Predicting Offset of Propofol’s Effects

Infusion regimens reflect the maturation of clearance, and practitioners should be cognizant of adverse effects from concentrations greater than the target plasma concentration. Decrement times correlate with the time taken to regain consciousness. The most widely recognised decrement time, the context-sensitive half time, is the time for plasma concentration of anesthetic agents to decrease by 50% (CSHT). It correlates poorly with the time taken to regain consciousness because the decrement time to consciousness may not be 50%. The return to consciousness correlates better with a concentration in an effect compartment (Ce) rather than plasma concentration. The delay between plasma and effect compartments is described by an equilibration half-time, although this is short for propofol T_1/2_keo 2.38 min [79].

An alternative decrement time, CST_AWAKE_, is the time required for propofol to decrease from a steady-state concentration to a predetermined effect compartment concentration associated with movement (e.g., 2 mg/L) which has been described [80]. An understanding of this propofol decrement time can be used to guide recovery after anaesthesia. A simulation was used to determine the time for the concentration to decrease from steady-state at a typically targeted effect compartment concentration of 3.5 mg/L (µg/mL) in children [80]. These times were short and reflected a decrement time to consciousness (CST_AWAKE_) that plateaued with longer infusion times. CST_AWAKE_ ranged from 7.5 min in a 1-year-old infant given propofol for 15 min to 13.5 min in a 15-year-old adolescent given a 2-h infusion. Neonates had prolonged increment times because the clearance was immature. The CST_AWAKE_ was 10 min after 15 min infusion and 18 min after 120 min infusion using a target concentration of 3.5 mg/L (µg/mL). Use of a higher target concentration of 6 mg/L (µg/mL) doubled decrement times. Delayed recovery beyond these simulated CST_AWAKE_ times is likely more attributable to the use of adjuvant drugs or the child’s clinical status.

### 5.4. The Loading Dose of Dexmedetomidine

Dexmedetomidine is a drug with sedative, anxiolytic, sympatholytic and analgesic properties [81]. A concentration-response relationship for sedation has been described in adults [60,61,82]. The central alpha-2 effects of the drug are also associated with the adverse effects of hypotension and bradycardia [83], while direct peripheral vasoconstrictor effects cause hypertension [84]. Rapid intravenous delivery of drugs will achieve a target concentration for sedation at the expense of adverse effects.

Classical teaching is that the dose can be calculated for a one-compartment model by simply using the volume of distribution (V) and target plasma concentration (Cp_TARGET_).

(7) $$\mathrm{Dose}=V\;\times {\;\mathrm{Cp}}_{\mathrm{TARGET}}$$

This calculation may not be applicable to many anaesthetic drugs that are characterised using multi-compartment models rather than a 1-compartment model. The drug itself can have physiological effects (e.g., cardiac output, peripheral vasoconstriction or organ blood flow changes) that alter drug distribution. Dexmedetomidine has a direct vasoconstrictor effect [85] that may reduce central compartment volume estimation [84,86].

It is assumed that this central volume plays an important role in loading dose determination. Use of an estimated volume that is small (e.g., V1 of 1.78 L/70 kg) [87] constructs a smaller loading dose than a larger volume estimate (e.g., V1 > 25.2 L/70 kg) [83,88,89] [17,90,91]. The use of an inappropriate dexmedetomidine central volume could lead to plasma concentrations that either cause adverse effects or inadequate sedation. However, the V1 parameter alone does not dictate the loading dose. Simulation of loading dose using reported PK parameter estimates and adverse effects models improves loading dose calculation and the need for that loading dose to be delivered slowly.

The central volume parameter V1 is unsuitable for calculating loading dose due to uncertainty around its estimation, multi-compartment kinetics, and the temporal delay as the drug distributes from plasma to the effect site. The use of slow infusion to deliver a dexmedetomidine loading dose was clinically determined and avoided adverse effects. It is not solely the central volume (V1) that determines the dose when a drug is given slowly.

The other pharmacokinetic parameters, aside from V1 (i.e., CL, V2, Q2), that are used to describe disposition influence dose determination. The loading dose should be used to target a concentration at the effect site. There is a time delay between peak plasma concentration and peak concentration at the effect site. The vasoconstrictor effect is immediate and does relate closely to plasma concentration, but the dexmedetomidine equilibration half-time (T_1/2_keo) is 3–6 min for sedative effect [60,61] and 9.9 min for vasodilation mediated centrally [84].

The time to peak effect (T_PEAK_) is dependent on clearance and the effect-site equilibration half-time. At a submaximal dose, T_PEAK_ is independent of dose. At supramaximal doses, the maximal effect will occur earlier than that T_PEAK_ predicted by the submaximal dose. The maximal effect persists for a longer duration because of the shape of the pharmacodynamic (PD) concentration-response relationship, described by the Hill equation.

Time-to-peak effect modelling has been used to calculate the optimal loading dose for a target concentration in the effect compartment [92]. The volume of distribution (Vpe) at the time of peak effect-site concentration (C_PEAK_) can be calculated.

(8) $$\mathrm{Vpe}=\frac{\mathrm{Dose}}{\mathrm{Concentration}\left(T_{\mathrm{PEAK}}\right)}$$

Loading dose can then be determined.

(9) $$\mathrm{Loading}\;\mathrm{Dose}=C_{\mathrm{PEAK}}\times \;\mathrm{Vp}$$

**Figure 2:**
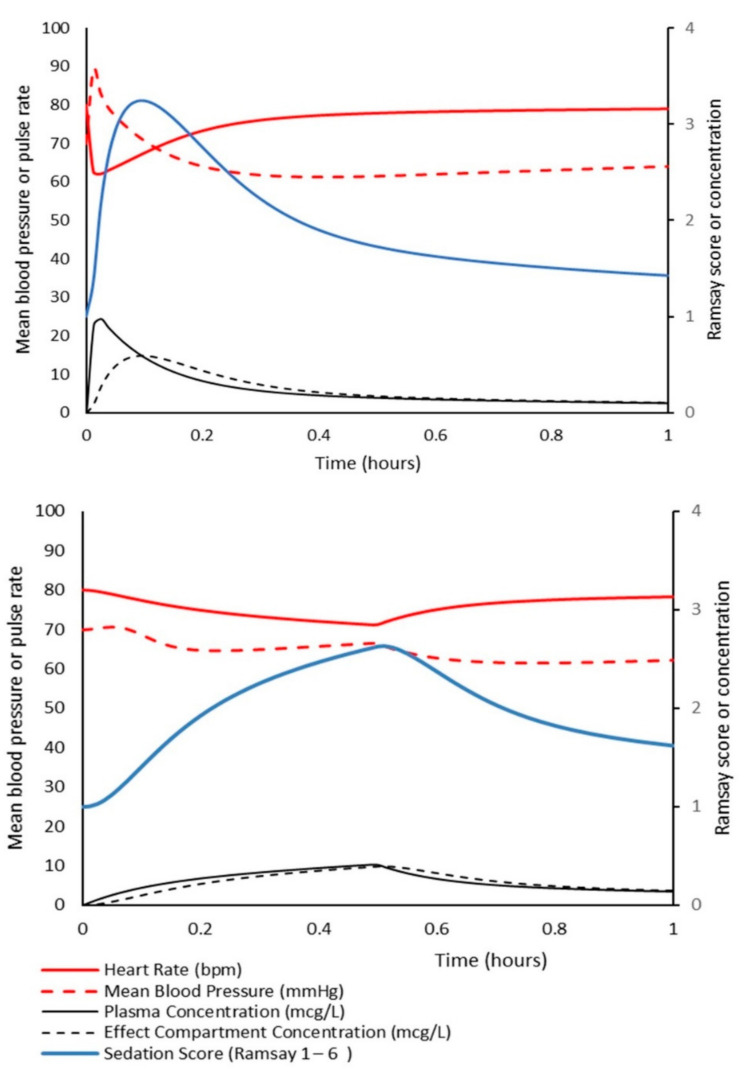
A simulation of dexmedetomidine demonstrating plasma concentration and haemodynamic effects when given intravenously to a 5-year-old child. The upper panel shows effects when the drug was given as a rapid intravenous bolus of 0.49 µg/kg. The lower panel shows concentration and haemodynamic changes after a larger dose of 0.57 µg/kg was administered as an infusion over 15 min. A similar level of sedation is achieved at 15 min, but slow infusion does not have the same magnitude of blood pressure and heart rate changes observed with rapid infusion.

The simulations in Figure 2 can be used to determine that the dexmedetomidine volume of distribution at the time of peak effect-site concentration (Vpe) was 0.38 L/kg when dexmedetomidine 0.49 µg/kg was given rapidly over 5 s; this represents a Vpe that approximates V1. However, when dexmedetomidine was administered over 15 min, a larger Vpe of 0.74 L/kg was necessary. An infusion over 15 min allows time during administration for redistribution of the drug, and V1 consequently has less importance; all parameter estimates (CL, V1, Q2, V2, Q3, V3) have an influence on observed plasma concentration.

### 5.5. Drug Interactions

Drugs are rarely given alone to children undergoing anaesthesia. The clinical endpoints required for surgery (unconsciousness, analgesia and loss of responsiveness to noxious stimuli) may correlate with many drugs’ upper tolerated dose range. Consequently, anaesthetic, neuromuscular and analgesic drugs are combined at lower doses so that the desired effects are compounded and unwanted drug effects can be lessened. The inclusion of drug interactions in pharmacokinetic-pharmacodynamic (PKPD) models increases their clinical applicability and usefulness and can provide the opportunity to describe the time course of multiple drug effects [93].

#### 5.5.1. Understanding Remifentanil Contribution to Bispectral Index

The bispectral index is a processed electroencephalogram that guides the drug dose for the depth of anaesthesia. The BIS monitor is agent-specific. Opioids have little impact on BIS. This is because the monitor provides information mainly limited to cortical brain activity that reflects the hypnotic component of anaesthesia. The analgesic component of anaesthesia involves subcortical autonomic responses not detectable by the BIS.

An additive propofol-remifentanil interaction model estimated a propofol C_50_ 3.99 mg/L (µg/mL) with a high remifentanil C_50_ of 21 μg/L [79]. Similar estimates in healthy adult volunteers are reported; an additive interaction between propofol and remifentanil reported mean C_50_ estimates for propofol and remifentanil of 4.47 mg/L and 19.3 μg/L, respectively. These results confirm that the measured effect of remifentanil on BIS is minimal at clinically important doses in both adults and children. These results are also consistent with the premise that drug responses of children (but not of neonates and infants) are similar to those of adults [94]. It is not that remifentanil is ineffective, but rather that the measurement of analgesic effect is poor.

Parameter estimates from a similar study in adult volunteers were similar; the propofol C_50_ estimate was 4.47 mg/L, and remifentanil C_50_ was 19.3 µg/L [95]. However, the pharmacodynamic interaction between sevoflurane and remifentanil was synergistic for both the hypnotic and the analgesic components of anaesthesia and with a lower C_50_ for remifentanil when combined with sevoflurane [96]. The response was monitored using observational scales (verbal response, eye-opening, grimacing, coughing, withdrawal, or any other purposeful or non-purposeful movement). These results support the notion that the analgesic component of anaesthesia involves subcortical autonomic responses not detectable by the BIS.

#### 5.5.2. Midazolam and Protease Inhibitors

The protease inhibitor ritonavir has therapeutic benefits against the human immunovirus (HIV). Nirmatrelvir has recently been introduced for the early management of proven COVID-19 infection in the community [97]. Nirmatrelvir is combined with a protease inhibitor, ritonavir, that slows the metabolism of nirmatrelvir via cytochrome P-450 (CYP3A) enzyme inhibition. Ritonavir will reduce the clearance of other drugs metabolised by the CYP3A enzyme system if used in the perioperative period. It is reasonable to allow ritonavir 4–5 half-lives to elapse for the clinical effect of this interaction to become minimal. Clearance reduction of other drugs could then be assumed present from 24 h after starting and 24 h after ceasing ritonavir.

Midazolam is one such drug metabolized mainly by hepatic hydroxylation (CYP 3A4) [98]. The inhibition of this enzyme in the gut by ritonavir increases bioavailability, while inhibition in the liver slows clearance [99]. The use of modelling can demonstrate the anticipated effects in children. PKPD relationships have been described for IV midazolam in adults. EEG signals that were quantified by the use of fast Fourier transformation were used as a response measure. The averaged amplitudes (square root of power) in the 11.5 to 30 Hz frequency band (beta) of each recording session were calculated and used as an EEG effect measure in the kinetic-dynamic modeling procedure.

**Figure 3:**
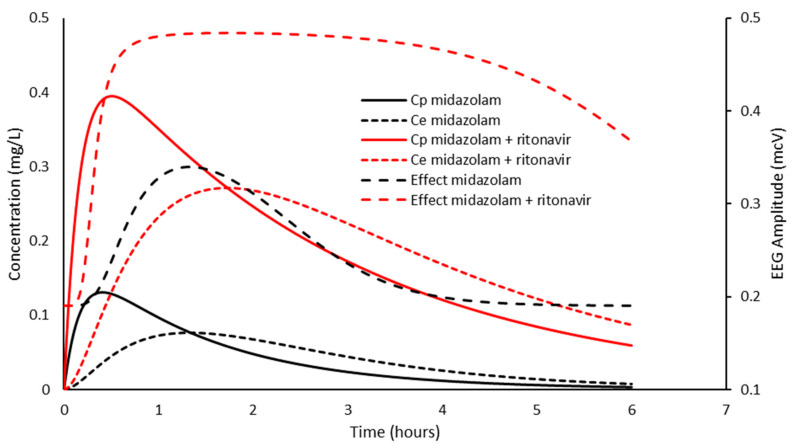
A child was given midazolam 0.5 mg/kg orally. The effect compartment concentration (Ce) is linked to plasma concentration by a rate constant (keo). The simulation shows the impact of increased bioavailability (2.7-fold) and slower clearance (assumed 50% reduction). Electroencephalographic amplitudes in the 11.5 to 30 Hz (beta) frequency band were used as an effective measure. Not only is the electroencephalographic effect prolonged due to slower clearance, but a ceiling effect is also achieved because of higher concentrations. PKPD parameter estimates from Mandema et al. [100].

When this processed EEG signal amplitude is used as an effect measure, the PD parameter estimates were: E0 0.19 mcV, Emax 0.3 mcV, C_50_ 77 mg/L, and N 3.1 with a T_1/2_keo of 1 min [100]. Figure 3 shows the impact of increased bioavailability (assumed 2.7-fold) [99] after oral administration.

The electroencephalographic effect indicative of sedation is prolonged due to slower clearance. A ceiling effect is also achieved because of higher concentrations that attain the ceiling effect. Sedation recovery relates to effect-site concentration and lags behind the decline in plasma concentration. An understanding of these effects obligates the use of a smaller oral dose and sedation monitoring before and after anesthesia in children with COVID-19 given ritonavir for treatment.

### 5.6. Acute Pain Management

The tenet that PKPD models can be used to determine a dose that will achieve a target concentration can also be used for opioids. That target concentration achieves a target effect. The target effect of opioids remains elusive because pain is such a personal experience. The use of surrogate measures of pain, such as pupillometry, has only a loose correlation [101,102].

#### 5.6.1. Morphine

A target concentration of 10 µg/L may be used for morphine analgesia. A concentration-analgesic response relationship has not been described [103], but this concentration is clinically effective for moderate pain and is lower than concentrations associated with respiratory depression [104]. Observations in children after cardiac surgery found that steady-state serum concentrations greater than 20 µg/L resulted in hypercarbia (Paco_2_ >55 mm Hg) and flattened CO_2_ response curves. Morphine concentrations above 15 µg/L caused hypercarbia in 46% of children. Lower concentrations below 15 µg/L were associated with hypercarbia in only 13% of children. This respiratory depression was concentration-dependent. The dose, determined using pharmacokinetic information and the covariates of age and size, is used to achieve a target concentration. Age and weight have little impact on the respiratory effect that occurs at the same serum concentration of morphine [105]. The initial target concentration may be 10 µg/L, but an observation or self-reporting pain scale is used at the bedside as part of a feedback loop for incremental dose changes.

#### 5.6.2. Oxycodone

No standard concentration-response relationship for oxycodone analgesia has been described in humans. However, a minimum effective concentration (MEC) determined by the need for intravenous rescue analgesia, and minimum effective analgesic concentration (MEAC), estimated by the relief of pain achieved after rescue analgesics [106,107], have been used as oxycodone target concentrations.

These concentrations differ with the type of pain. Interpretation of analgesia results is confusing. An oxycodone MEC 20–35 µg/L and MEAC 45–50 µg/L for pain after laparoscopic cholecystectomy in 23 adult patients has been described [108]. We might have anticipated the higher mean MEC of 31 µg/L and MEAC of 75 µg/L reported in adults undergoing major intra-abdominal surgery [109]. However, sternotomy after cardiac surgery is expected to be painful and lower values were reported after adult cardiac surgery (MEC 6–12 µg/L MEAC 15–25 µg/L) [110]. The general therapeutic range of oxycodone is between 10–100 µg/L; this is too broad to be useful in defining a target concentration [108,109,110,111,112,113]. A specific target concentration that could be used to inform dosing remains elusive.

Oxycodone is associated with serious adverse effects (respiratory depression, nausea, itch, urinary retention) that are common to most opioids and are often related to concentration [114,115]. The target concentration of oxycodone should provide effective analgesia without increased risk of adverse effects. The time-course of respiratory depression in six healthy adult participants (21–30 years of age, 68–80 kg) given intravenous oxycodone (loading dose 50 µg/kg with 275 µg/kg/h infusion) and intravenous morphine (loading dose 39 µg/kg bolus with 215 µg/kg/h infusion) over 2 h were similar [116]. Fatal consequences are only reported with oxycodone concentrations greater than 200 µg/L [115], allowing a 3-fold margin of safety between MEC or MEAC values and oxycodone’s major adverse effect.

The use of a “best guess” target concentration of 35 mcg/L, based on morphine comparison, MEAC information and toxicity concerns, enabled dosing prediction using reported pharmacokinetic parameter estimates. The intravenous loading and maintenance doses for a typical 5-year-old child are 100 µg/kg and 33 µg/kg/h. In a typical adult, the loading dose is 100 µg/kg, and the maintenance dose is 23 µg/kg/h [16,117].

### 5.7. Models for Education

Pharmacokinetic modelling and simulation can facilitate the understanding and prediction of exposure-response relationships in children with acute or chronic pain. Diamorphine (diacetylmorphine, heroin) is a strong opioid with poorly described pharmacokinetics. Parameter estimates in children remain poorly quantified, and the dose is guided by clinical experience and analgesic effect comparisons with other opioids.

Diamorphine has complex clearance pathways that produce three active metabolites acting on both dopamine and opioid receptors. There is a need to better understand these diamorphine metabolites, time-variable effects and dose requirements of this drug in children. A model to describe diamorphine pharmacokinetics has been fashioned from a model for diamorphine in adults [118] and a model describing morphine and its metabolite disposition in children [12] to determine dose after intranasal or intravenous use in children. Allometric scaling and maturation models were applied to clearances and volumes to account for differences in size and age between children and adults.

**Figure 4:**
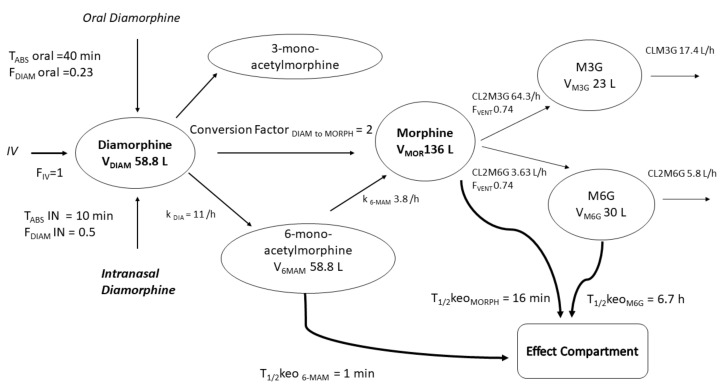
This diagram shows the metabolite flow of diacetylmorphine, 6-mono-acetylmorphine, morphine and morphine glucuronide metabolites. Diamorphine absorption is described in terms of absorption half-times (T_ABS_) and relative bioavailability (F_DIAM_) by oral or intranasal (IN) routes. Rate constants (k_DIA_, k_6-MAM_)) describe the flow between metabolites. Clearance of morphine 3-glucuronide (M3G) and morphine 6-glucuronide (M6G) are driven by renal function. The delay between active metabolites (6-MAM, morphine, M6G) and the effect compartment is described using equilibration half-times (T_1/2_keo).

The model (Figure 4) explained that reported observations can be used for interrogation, interpolated to determine equianalgesic and inform future clinical studies.

**Figure 5:**
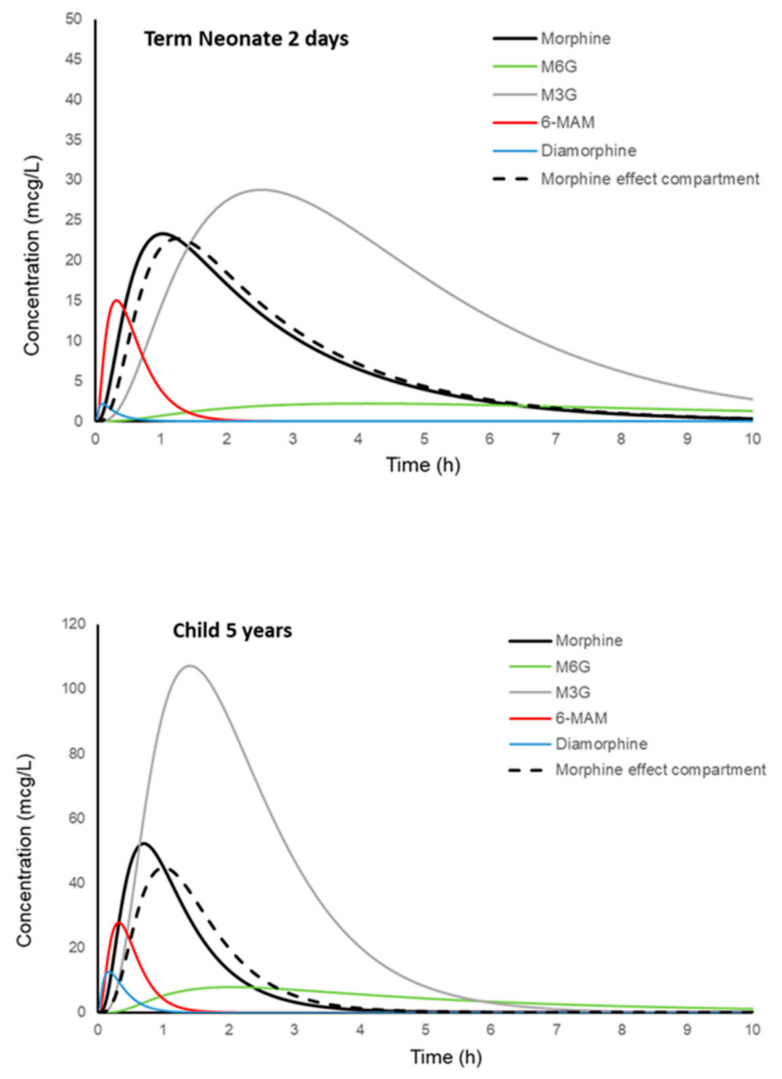
Simulated time-concentration profiles for diamorphine and its metabolites are shown for a neonate (3.2 kg, PNA 2 days, 40 weeks PMA) and a child (5 years, 20 kg) given intranasal (IN) diamorphine. A neonate given diamorphine 33 µg/kg IN achieved a similar area under the morphine curve (AUC_0–10_) to a child given 98 µg/kg IN or an adult given 70 µg/kg IN (5 mg). Morphine peak concentrations (C_MAX_) are lower in neonates than in older children, but concentrations are above 10 µg/L for a longer duration. A 5-year-old child has a bigger C_MAX_ but a shorter duration of exposure.

A simulation was used to illustrate how diamorphine is rapidly metabolised to morphine through the active metabolite 6-monoacetylmorphine (6-MAM). This 6-MAM metabolite initiates an early dopaminergic response that contributes to euphoria immediately after intravenous administration. Morphine formation is then responsible for the slower, prolonged analgesic response. The active gluconiated metabolite of morphine (M6G) accumulates in renal failure and exacerbates respiratory depression [119]. Time-concentration profiles of diamorphine and its metabolites change with age and reflect disposition changes as neonates mature into children. These profiles (Figure 5) described intravenous and intranasal dosing regimens. These indicated that morphine exposure in children after intranasal diamorphine 0.1 mg/kg was similar to after intranasal diamorphine 5 mg in adults [120].

Interaction displays such as GE Navigator (GE Healthcare, Helsinki, Finland) and SmartPilot View (Drager Medical, Lubeck, Germany) can be used as an educative tool for visualising individual PKPD models and drug interactions. These are currently only available with adult models, but it is hoped such displays may become available with paediatric models in the near future. These are of clinical use for monitoring the adequacy of anaesthesia. Both displays can optimise the administration and monitoring of anaesthetic drugs during general anesthesia and may reduce the consumption of volatile anaesthetic agents [121].

A Shiny App is another tool that can be used for educational purposes. It is possible to program interactive PKPD models with a Shiny Web-browser interface that can be viewed on the user’s own computer or on another computer accessed by means of the Internet [122].

## 6. Conclusions

Pharmacokinetic-pharmacodynamic models have led to better drug delivery systems and parameter sets that can be used in TCI pumps for total intravenous anaesthesia. Some of these new PKPD models can be used in ages from neonates to elderly adults and across individuals with a wide range of weights. A simulation using these models in children has provided guidance on drug offset when input is stopped. Dose in any delivery system is tempered by adverse effects. Hypertension associated with a loading dose of dexmedetomidine or remifentanil can be tempered by slowing the infusion rate. Inhalational agents conform to compartment models, allowing the investigation of drug interactions with other sedatives such as propofol; the propofol dose required to prevent awareness during the transition from inhalational anaesthesia to TIVA has been quantified. Quantification of drug interactions has also improved understanding of the effects of drug mixtures. Investigation of propofol/remifentanil interaction revealed a high remifentanil C_50_; the drug is an ineffective sedative but synergistically influences other hypnotics and sedatives. Remifentanil’s sedative effect is also poorly reflected by the bispectral index or other current processed EEG monitoring. Repurposed drugs (e.g., antiviral drugs used for COVID-19) used within the community can have important effects on drugs used in paediatric anaesthesia, and the use of simulation educates about drug vagrancies. Some drugs exert an effect through a confusing spectrum of metabolites. The use of a model can be educative, improving understanding of the drug, delivery and indications.

## Data Availability

Not applicable.
